# Metals and metalloids in high-altitude Pyrenean lakes: sources and distribution in pre-industrial and modern sediments

**DOI:** 10.1007/s11356-023-28347-6

**Published:** 2023-07-10

**Authors:** Azibar Rodriguez-Iruretagoiena, Ainara Gredilla, Silvia Fdez-Ortiz de Vallejuelo, Gorka Arana, Maite Meaurio, Juan Manuel Madariaga, Jean Christophe Auguet, Aridane González González, Oleg S. Pokrovsky, Luis Camarero, Alberto de Diego

**Affiliations:** 1 Department of Analytical Chemistry, University of the Basque Country (UPV/EHU), 48940 Leioa, Basque Country Spain; 2 Hydrogeology and Environment Group, Science and Technology Faculty, University of the Basque Country (UPV/EHU), 48940 Leioa, Basque Country Spain; 3 Marine Biodiversity, Exploitation and Conservation (MARBEC), Université de Montpellier, CNRS, IFREMER, Montpellier, France; 4 Géosciences Environnement Toulouse (GET) – Research institute for development [IRD]: UMR239, Paul Sabatier Unibersity [UPS] - Toulouse III, CNRS: UMR5563, Toulouse III, France; 5 Instituto de Oceanografía y Cambio Global, IOCAG, Universidad de Las Palmas de Gran Canaria, ULPGC. Parque Científico Tecnológico Marino de Taliarte, s/n, 35214 Telde, Las Palmas, Spain; 6 BIO-GEO-CLIM Laboratory, Tomsk State University, 36 Lenina Prs, Tomsk, 630050 Russia; 7 Advanced Studies Center of Blanes (ceab), C/ d’accés a la Cala St. Francesc, 14. Blanes, E-17300 Girona, Spain; 8 Research Centre for Experimental Marine Biology and Biotechnology (PIE), University of the Basque Country (UPV/EHU), P.O. Box 644, 48080 Bilbao, Spain

**Keywords:** Sediment core, High-altitude lakes, Natural, anthrophonic, Pyrenees, Metals and metalloids

## Abstract

High-altitude Pyrenean lakes are ecosystems far from local pollution sources, and thus they are particularly sensitive to the atmospheric deposition of metals and metalloids. This study aims to quantify the effect of human activity in 18 lakes located in both side of the France–Spain frontier. Sediment cores were collected in summer 2013, sampled at a 1cm resolution and the concentration of 24 elements was measured by ICP-MS. Statistic and chemometric analysis of the results highlights the influence of the geographical position and lithogenic features of each lake basin on trapping pollutants. More than the 80% of the lakes showed values of enrichment factor (EF) above 2 for at least one of the elements investigated in at least one core interval, which corroborates the existence of historical anthropogenic inputs of elements in the studied area. The results demonstrate the natural origin of As and Ti in Pyrenees, together with the significant anthropogenic inputs of Cd, Pb, Sb and Sn from ancient times. The data set points mining activities as the main historical source of pollution and illustrate the large impact of the industrial revolution. The regional variability could reflect also differential long-range transport, followed by dry or wet deposition.

**Supplementary Information:**

The online version contains supplementary material available at 10.1007/s11356-023-28347-6.

## Introduction

Human activity has an irrefutable effect on climate and on the biogeochemical cycles of major and trace elements (IPCC 2022; Moser et al. 2019; Nriagu 1996). The anthropogenic impact is not only perceived at a planetary scale, but also at a regional or even local scale. The Pyrenees lie on the transition zone between the Atlantic and the Mediterranean climatic regions. This fact, combined with the unique geographic and climatic features of mountains, makes the Pyrenees especially sensitive to global climate effects in SW Europe, an area where large rates of climate change are expected. Industrial and urban activities in the surrounding cities, such as Bilbao, Bordeaux, Zaragoza, Toulouse and Barcelona, can affect the quality of its ecosystems in the short term and the long history of human activities may have a long-term impact too. However, because of administrative and political borders, the Pyrenean area is divided into three countries (France, Andorre and Spain), and that has limited significantly the amount of research that has been done considering the area as a whole.

The Pyrenees mountain range extends from Matxitxako Cape (Basque Country) to Creus Cape (Catalonia), reaching its highest point at about 3400 m (Aneto peak, Aragon). Some excellent examples of sensitive ecosystems in Pyrenean region are high altitude (<2000 m), where the presence of pollutants is usually small, and thus they are the best sentinels to identify the effects of contamination in the environment (Bacardit and Camarero 2010a; Camarero 2003;). Within the lake system, sediments are more conservative than water, and contain an invaluable record of pollutants all over time, which makes them an essential matrix for environmental studies (Ruiz-Fernández et al. 2003).

The oldest long-range atmospheric pollution is that of metals and metalloids. It started more than two millennia ago (Camarero 2017). Consequently, sediments have been widely used to reconstruct metalworking activities all around the world, making possible the differentiation between “older” and “modern” metal contamination (Heim and Schwarzbauer 2013).

Despite metals and metalloids are important as micronutrients, they have negative toxic effects (Nriagu and Pacyna 1988). From a toxicological point of view, their persistence to biodegradation and their accumulation capacity are the main characteristics (Zhang and Gao 2015). As an example, Cd, Hg, Ni and Pb are part of the priority toxic substances list of *The European Water Framework Directive, (EWFD)* (WFD, 2022). Many other elements, such as As, Cr, Cu and Zn, are ranked among the priority metals with public health significance. Some of these metallic elements are considered systemic toxicants that are known to induce multiple organ damage, in some cases even at low levels of exposure (Naimo 1995).

Regardless their toxicological risk, it is important to consider that metals have both natural and anthropogenic origin. With respect to natural sources, they are primary constituents on the Earth’s crust; consequently, their presence in air, rock, soil, sediments and biota varies across geographic regions. As part of the natural biogeochemical cycle, metals are released from rocks by weathering processes. Some other natural sources include volcanic activity and forest fires (Zuski et al., 2007). However, anthropogenic inputs alter the mentioned natural cycle of metals, increasing their concentration, and contributing to their accumulation. Agricultural, industrial or mining activities, together with road traffic are the principal metal sources. Once they are released in the environment, they can be distributed as direct discharge or by atmospheric transport. In the atmosphere, metals mostly appear adsorbed to particles, which can be long-range transported from their starting source, before being deposited again (Patterson and Settle 1987) to end up accumulated at lakes sediments. Wind direction and intensity, together with mountain orography and lake altitude (among others), have a direct effect on the long-range transport of metallic (and others) pollutants through the atmosphere (Bacardit 2011; Camarero 2013), and its further wet or dry deposition (Marques et al. 2004).

Metal deposition has been described not only in Pyrenees (Camarero et al., 2009), but also in alpine lakes all over the globe (Catalan et al. 2013). One of the clearest example we have are the Saharan dust outbreaks, which are important natural inputs of a wide range of pollutants to extensive areas of the world (Escudero et al. 2005), acting as a carrier of an enriched cocktail of diverse metals and metalloids. However, not all mountain lakes respond in the same way or at the same time to pollution episodes and/or climate changes due to internal lake processes (Baron and Caine 2000). Thus, studies carried out at a regional scale and considering both surface and older sediments are mandatory. More studies that include a sufficient number of lakes are needed to be able to represent the geographic variability of the area under study, in terms of location, altitude and lithology, and researches that comprise the analysis of metals and metalloids of different nature and origin. These studies may integrate all the processes and interactions that occur in a lake basin in order to establish metal backgrounds, footprints and tendencies of a sensitive area, such as the Pyrenees mountain range.

The aim of this study is to investigate the occurrence and distribution of 24 metals and metalloids in surface and deep sediments of Pyrenean high-altitude lakes located in both sides of the border (France and Spain). A similar study was conducted several decades ago (Bacardit 2011; Camarero 2003; Camarero et al. 2009) and included the measurement of the concentration of As, Cd, Cu, Hg, Pb, Se and Zn in lake sediments collected in around 75 different lakes (located in both sides of the border, above the local tree line and with more than 0.5 Ha size). Our work tests the conclusions obtained by previous surveys and extent the analyses to a larger number of elements to investigate metal and metalloid pollution occurrence and distribution.

The use of statistics and chemometric techniques allowed us to (i) study the geographical distribution of metals and metalloids throughout the Pyrenees, (ii) investigate historical records and identify past peaks of pollution and (iii) discern between natural and anthropogenic inputs of pollutants.

## Material and methods

### Sampling and sample pretreatment

**Fig. 1:**
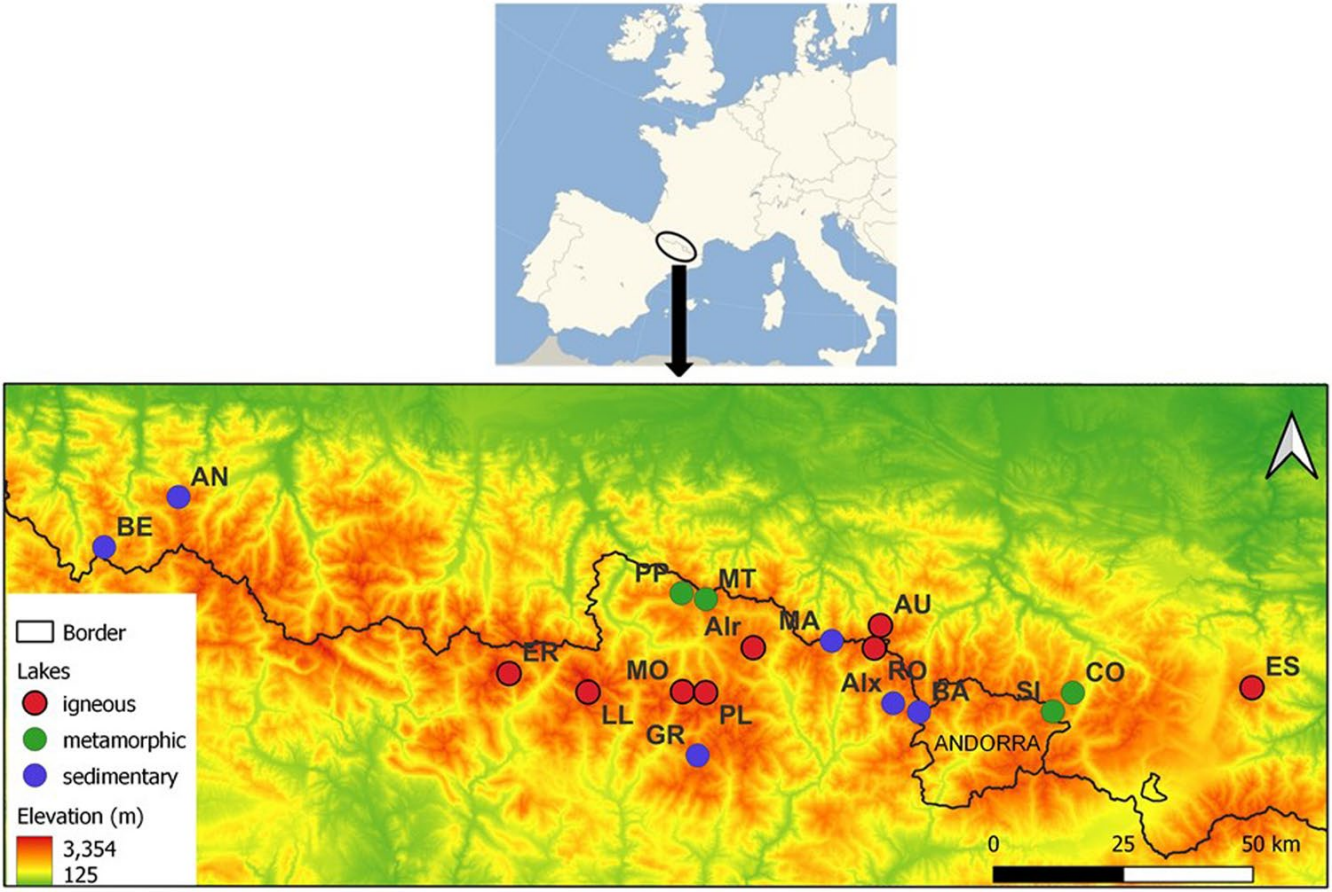
Localization of the 18 high-altitude lakes studied in Pyrenees coloured according to their major geology, in alphabetical order and situated in a digital elevation model map: Airoto (AIr), Aixeus (AIx), Anglas (AN), Aubé (AU), Baiau (BA), Bersau (BE), Compte (CO), Eriste (ER), Estelat (ES), Gran del Pesso (GR), Llosas (LL), Mariola (MA), Monges (MO), Montoliu (MT), Pica Palomera (PP), Plan (PL), Romedo de Dalt (RO) and Siscar (SI)

A single sampling campaign was performed in Summer 2013 at 18 high-altitude Pyrenean lakes (>2000 m; Fig. 1), located in France (Anglas, AN; Aubé, AU; Bersau, BE; Compte, CO; Estelat, ES; and Siscar, SI) and Spain (Airoto, AIr; Aixeus, AIx; Baiau, BA; Eriste, ER; Gran del Pesso, GR; Llosas, LL; Mariola, MA; Monges, MO; Montoliu, MT; Plan, PL; Pica Palomera, PP; and Romedo de Dalt, RO). The geographical location and some geomorphological characteristics of these lakes are provided in Fig. 1 and Table 1 and (adapted from del Castillo 2004). Igneous and metamorphic rocks are the most extended lithology in the central and eastern Pyrenees, but in the central and western areas, carbonate formations also occur (Garcia-Pausas et al. 2007). As it can be observed in Table 1, igneous rocks, and specifically acidic plutonic rocks, are abundant within the lakes studied. The sedimentary rocks in which the lakes are located are mainly shales, sandstones and conglomerates, while most of the metamorphic show regional metamorphism varying from low to high grade.

**Table 1:** The geographical location (UTM 31T) and several geomorphological characteristics (adapted from del Castillo, 2004) of the lakes included in this study, in alphabetical order. The total depth of the sediment core collected at each lake is also provided

Name	UTM 31T (*X*)	UTM 31T (*Y*)	Id	Size (ha)	Catchment (ha)	Perimeter (m)	Altitude (m)	Max Depth (m)	Lithology	Sed core (cm)
Airoto	339405	4729463	AIr	18.7	205	1964	2215	41	Igneous	5.0
Aixeus	366449	4718687	AIx	3.5	94	737	2368	16	Sedimentary	22.5
Anglas	722574	4753940	AN	2.8	110	615	2068	39	Sedimentary	12.0
Aubé	363971	4733677	AU	7.9	65	1139	2100	45	Igneous	19.0
Baiau	371347	4716960	BA	7.8	140	1376	2485	22	Sedimentary	26.0
Bersau	704135	4748827	BE	11.8	58	1740	2080	33	Sedimentary	12.0
Comte	401039	4720626	CO	3.4	929	873	1726	5	Metamorphic	22.5
Eriste	292437	4724450	ER	0.8	(-)	440	2454	21	Igneous	26.5
Estelat	435526	4721626	ES	4.4	161	1070	2022	4	Igneous	36.5
Gran del Pesso	328762	4708588	GR	9.1	(-)	1347	2490	38	Sedimentary	5.0
Llosas	307658	4720810	LL	4.2	297	755	2480	32	Igneous	5.0
Mariola	354601	4730744	MA	17.5	121	1830	2270	46	Sedimentary	26.0
Monges	325896	4720922	MO	14.7	107	1917	2422	51	Igneous	5.0
Montoliu	330366	4738774	MT	10.7	117	1563	2375	17	Metamorphic	5.0
Pica Palomera	325700	4739902	PP	4.9	61	929	2291	9	Metamorphic	22.5
Plan	330298	4720754	PL	5	16	944	2190	10	Igneous	30.0
Romedo de Dalt	362790	4729315	RO	11.7	277	1769	2105	40	Igneous	36.5
Siscar	397224	4717099	SI	4.5	294	1134	2187	4	Metamorphic	33.0

All the lakes are classified as small, deep and dimictic, with mixing periods after thaw in June and during autumn from October to December.

Sediment cores were collected from the deepest part of the lake using an inflatable boat by means of a gravity core sampler. The length of the cores ranged from 5 cm (Airoto, AIr, Gran del Pesso, GR, Llosas, LL, Monges, MO and Montoliu, MT) to 36.5 cm (Estelat, ES, and Romedo de Dalt, RO) (see Table 1). Each core was in situ sliced immediately after collection using clean plastic tools. Between layers, all the material was cleaned with Milli-Q quality water (18.2 MV cm, Milli-Q Element A10 purification system, Merck Millipore, Billerica, MA, USA). The surface slice 0–1.5 cm (surface layer) was first separated and stored in a clean pre-labelled zip bag. The next 3.5-cm slices were successively obtained and stored in a similar way. One hundred and eight samples were finally obtained for analysis. All of them were transported downhill in refrigerated backpacks to the laboratory. The sediment samples were kept frozen (−20°C) in the laboratory until freeze-drying in a Criodos apparatus for 72 h (−52°C, 150 mTorr, Telslar, Madrid, Spain). Dry sediment samples were sieved to assure a maximum particle size of 63 μm, and stored in the refrigerator at 4°C in darkness until analysis.

### Analysis

All glass material used during the analysis step was washed with tap water and soap and left in a 10% nitric acid (Panreac, Barcelona, Spain) bath for at least 24 h. Afterwards, it was thoroughly rinsed twice with Elix quality water (Merck Millipore, Billerica, MA, USA) and once with Milli-Q quality water, and stored in clean zip bags until use.

About 0.5 g (±0.0001 g) of dried and sieved sediments was weighed in a AJ150 balance (Mettler-Toledo S.A.E., Barcelona, Spain) and subjected to an extraction procedure using a Multiwave 3000 microwave oven (Anton Paar, Graz, Austria), with a maximum power output of 1400 W, and equipped with eight 100 mL fluorocarbon polymer (PTFE) microwave vessels in an 8XF-100 microwave digestion rotor. Eight samples were simultaneously digested during each run. The EPA 3051A method (USEPA 2007) was followed for the extraction procedure, using a mixture of 3 mL of HCl (36%; Tracepur grade, Merck Millipore, Billerica, MA, USA) and 9 mL of HNO_3_ (69%; Tracepur grade, Merck Millipore, Billerica, MA, USA) as extractant.

The obtained extracts were passed through 0.45 μm PVDF syringe filters (Olimpeak, Teknokroma, Barcelona, Spain), and quantitatively transferred into pre-cleaned polyethylene tubes. All the extracts were gravimetrically (±0.0001 g, Mettler-Toledo AJ150 balance) diluted with Milli-Q quality water, and their acidity was adjusted to 1% HNO_3_. The diluted samples were again accurately weighed and stored in darkness at 4°C until analysis.

The total concentrations of Ag, Al, As, Ba, Cd, Co, Cr, Cu, Fe, Hg, Mg, Mn, Mo, Ni, Pb, Sb, Se, Sn, Sr, Ti, Tl, V, W and Zn were measured in all the diluted extracts by inductively coupled–plasma mass spectrometry (ICP-MS) in a NexION 300X (Perkin Elmer Inc., Waltham, MA, USA) located inside a 100-class clean room conditioned at 20°C. More details about the experimental conditions used in the analysis can be found elsewhere (Liñero et al. 2017).

The trueness and precision of the method were checked by replicate analysis (*n* = 8) of the certified reference material NIST 1646a (estuarine sediment, National Institute of Standards and Technology). All the obtained concentrations (in mg·kg^−1^) were corrected taking into account the actual humidity content of the reference material. Satisfactory results were obtained for all the elements certified in the CRM (relative standard deviations below 9 % and recoveries between 77 and 105%), being the analytical method used suitable for our purpose (Table 2). Procedural blanks (*n* = 8) were also run in the same way to estimate the detection limits of the method (Table 2).

**Table 2:** Concentrations (in mg·kg^−1^) of metals and metalloids in surface sediments (0–1.5 cm) collected from 18 high-altitude Pyrenean lakes, together with some variable descriptive statistics. The table shows the detection limit (LOD), recovery and reproducibility of the analytical method using BCR 701 reference material (*n* = 8). Extreme high concentrations, defined as those concentrations over the 75th percentile of the data (Q3) plus three times the interquartile range (IQR), are marked in bold italics

	Ag	Al	As	Ba	Cd	Co	Cr	Cu	Fe	Hg	Mg	Mn	Mo	Ni	Pb	Sb	Se	Sn	Sr	Ti	Tl	V	W	Zn
LOD (μg·kg^−1^)	20	175	11	83	34	4	5	1	36	101	19	599	34	162	76	7	950	21	275	8	12	5	2	1
Recovery^1^ (%)					97		76	71						70	89									86
Reproducibility (%)	6	4	6	4	3	6	2	5	5	6	4	6	2	3	3	4	6	6	2	2	4	3	6	6
AIr	0.48	8260	***1470***	17.6	1.50	3.30	12.1	42.5	7660	0.28	1720	112	0.50	5.45	74.7	0.87	1.51	4.35	5.13	118	0.22	13.0	***4.96***	149
AIx	0.35	29600	31.5	46.6	0.46	7.38	20.4	73.3	92700	<LOD	6230	196	0.82	27.0	40.4	0.55	<LOD	1.29	9.11	405	0.052	21.2	0.048	110
AN	0.15	23100	382	61.0	***23.5***	15.3	36.9	21.9	39800	1.52	7240	463	0.95	34.4	209	1.21	5.18	3.13	30.5	726	0.15	41.7	0.36	***4690***
AU	0.27	30100	14.4	81.3	1.66	5.54	25.5	16.2	17800	0.19	5810	152	0.89	11.0	135	1.22	1.65	9.21	14.6	830	0.38	35.1	0.27	272
BA	1.13	41700	71.4	61.8	0.38	18.7	22.7	157	66400	0.12	4320	118	0.86	44.0	61.7	1.21	1.21	2.14	5.13	351	0.15	29.0	0.25	183
BE	0.63	36000	242	36.9	0.10	16.0	20.6	188	65500	0.22	4330	126	3.94	32.1	27.8	0.32	***24.2***	0.53	18.4	260	0.14	24.5	0.54	150
CO	0.061	10500	7.06	41.4	1.01	2.39	12.2	6.19	7940	<LOD	2010	107	0.24	5.90	25.1	0.12	0.99	2.28	7.02	365	0.23	11.6	0.30	77.3
ER	0.18	25300	181	80.9	0.64	9.38	33.6	19.7	34500	0.12	6300	258	2.42	15.0	100	0.85	0.99	2.86	9.42	644	0.40	43.2	2.60	105
ES	0.23	13100	8.70	125	0.22	3.67	12.4	11.7	11300	<LOD	3600	144	0.081	5.95	26.3	0.20	<LOD	3.53	23.3	1070	0.13	32.1	0.45	63.5
GR	0.082	20300	85.6	60.2	1.06	7.57	20.0	14.4	17400	<LOD	5130	198	1.94	9.49	55.5	0.42	<LOD	1.74	12.2	582	0.26	40.0	0.23	90.0
LL	0.12	22500	59.9	98.0	1.78	7.74	24.2	15.1	22900	<LOD	6340	331	3.76	11.0	106	0.73	<LOD	6.30	11.0	1060	0.45	35.3	1.99	165
MA	2.49	28700	8.78	99.9	1.01	12.0	33.0	46.1	25700	0.12	6750	232	0.57	27.9	199	1.58	1.68	5.92	11.5	274	0.27	38.8	0.34	188
MO	0.51	34100	6.22	98.1	1.03	8.12	33.2	24.6	19600	0.57	6230	161	0.87	14.5	64.0	1.55	2.03	3.02	16.4	645	***1.23***	44.6	1.15	138
MT	0.47	18700	79.1	30.9	***22.3***	7.12	24.8	88.9	80200	0.27	3100	142	***17.7***	22.7	***764***	***9.86***	2.31	1.70	14.7	86.3	0.24	56.4	0.12	***4320***
PL	***25.5***	20800	60.8	68.9	1.21	6.69	23.6	29.8	28600	0.38	4220	157	2.45	16.5	111	1.13	<LOD	3.44	15.8	413	0.28	37.5	0.51	199
PP	1.50	22000	52.7	31.5	***10.4***	2.42	25.8	253	75800	0.60	2450	71.3	***26.3***	10.2	395	***8.07***	4.80	1.85	39.2	65.4	0.37	72.7	0.10	***2820***
RO	0.32	31600	41.3	104	1.21	8.28	28.6	26.8	29600	0.29	4520	144	1.74	14.6	189	1.96	1.91	8.16	17.1	512	0.37	41.4	0.86	127
SI	0.12	12000	5.90	38.7	0.53	2.86	12.9	8.21	9050	<LOD	2320	79.9	0.31	8.84	38.9	0.18	<LOD	2.79	7.51	333	0.13	14.0	0.05	48.1
25th percentile (Q1)	0.14	17300	8.76	38.3	0.51	3.57	18.2	14.9	15900	0.14	2930	116	0.56	9.33	40.1	0.39	1.28	1.82	8.71	270	0.15	23.6	0.20	102
75th percentile (Q3)	0.75	30500	109	98.0	1.69	10.0	29.7	77.2	65700	0.52	6250	206	2.78	27.2	191	1.56	4.18	4.74	17.4	666	0.37	42.1	0.93	217
IQR (Q3-Q1)	0.61	13200	100	59.7	1.18	6.43	11.5	62.3	49800	0.38	3320	90.1	2.22	17.9	151	1.17	2.90	2.92	8.69	396	0.22	18.5	0.73	115
Q3+(3*IQR)	2.58	70100	410	277	5.23	29.3	64.2	264	215000	1.66	16200	476	9.44	80.8	644	5.07	12.9	13.5	43.5	1850	1.03	97.6	3.12	562
Median	0.33	22800	56.3	61.4	1.05	7.47	23.9	25.7	27100	0.28	4430	148	0.92	14.5	87.5	1.00	1.79	2.94	13.4	409	0.25	36.4	0.35	150
Average	1.92	23800	156	65.7	3.89	8.03	23.5	57.9	36200	0.39	4590	177	3.68	17.6	146	1.78	4.04	3.57	14.9	485	0.30	35.1	0.84	772
Standard deviation	5.92	9270	342	30.7	7.28	4.78	7.78	70.5	27400	0.19	1770	95.9	6.92	11.2	180	2.68	6.49	2.38	8.86	301	0.26	15.3	1.24	1500
Min	0.06	8260	5.90	17.6	0.10	2.39	12.1	6.19	7660	<LOD	1720	71.3	0.081	5.45	25.1	0.12	<LOD	0.53	5.13	65.4	0.052	11.6	0.048	48.1
Max	25.5	41700	1470	125	23.5	18.7	36.9	253	92700	1.52	7237	463	26.3	44.0	764	9.86	24.2	9.21	39.2	1070	1.23	72.7	4.96	4690

### Data processing

**Fig. 2:**
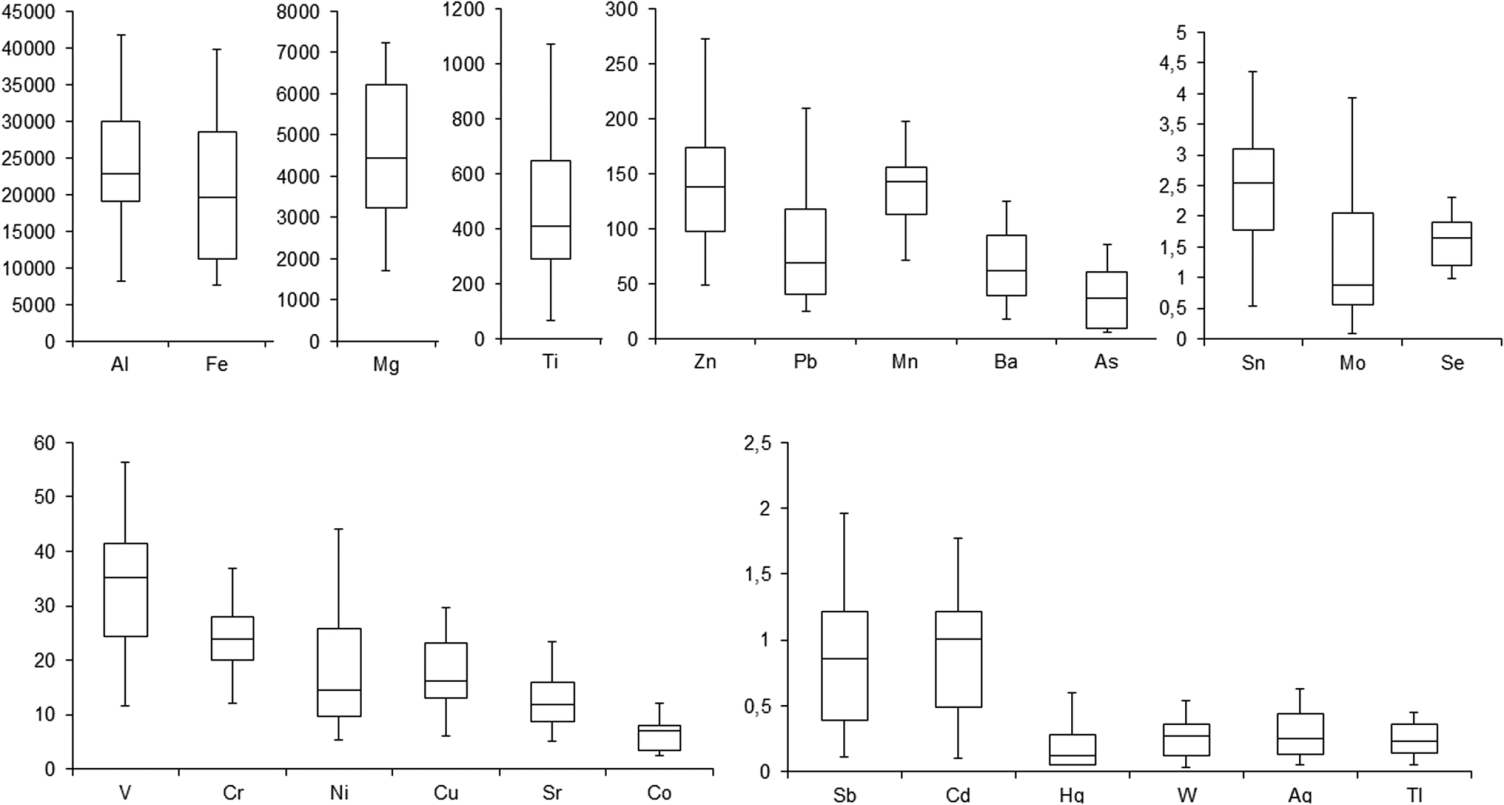
Element concentrations (in mg·kg^−1^) found in surface sediments from 18 high-altitude lakes of the Pyrenees. Box-Whisker plots have been calculated after removing all extreme (*c* > Q3+3*(Q3–Q1)) and outlier (*c* > Q3+1.5*(Q3–Q1)) values from data

**Fig. 3:**
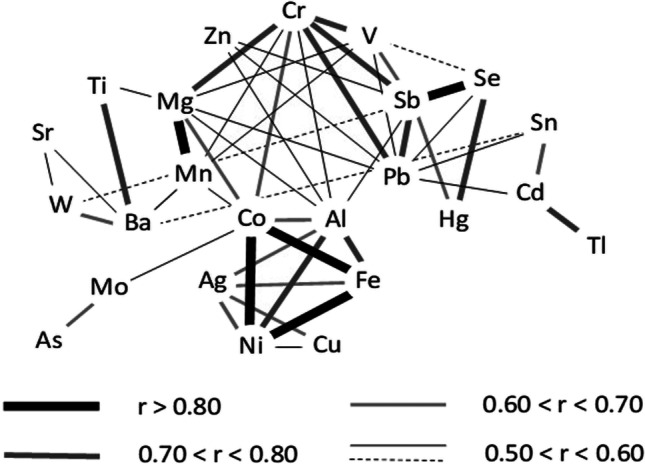
Correlation among pairs of elements obtained after removing extreme and outlier values from the dataset. Coefficients (*r*) higher than 0.49 are significantly different from zero at a 95% significance level

The Kolmogorov-Smirnov test demonstrated that the data were not normally distributed, and thus non-parametric statistics was used. The Kruskal-Wallis test was used to check the possible existence of significant differences between elements concentration in different lakes and different depths. The level of significance was fixed at 0.05 (95% confidence level) to consider a result as statistically significant (Figs. 2 and 3).

**Fig. 4:**
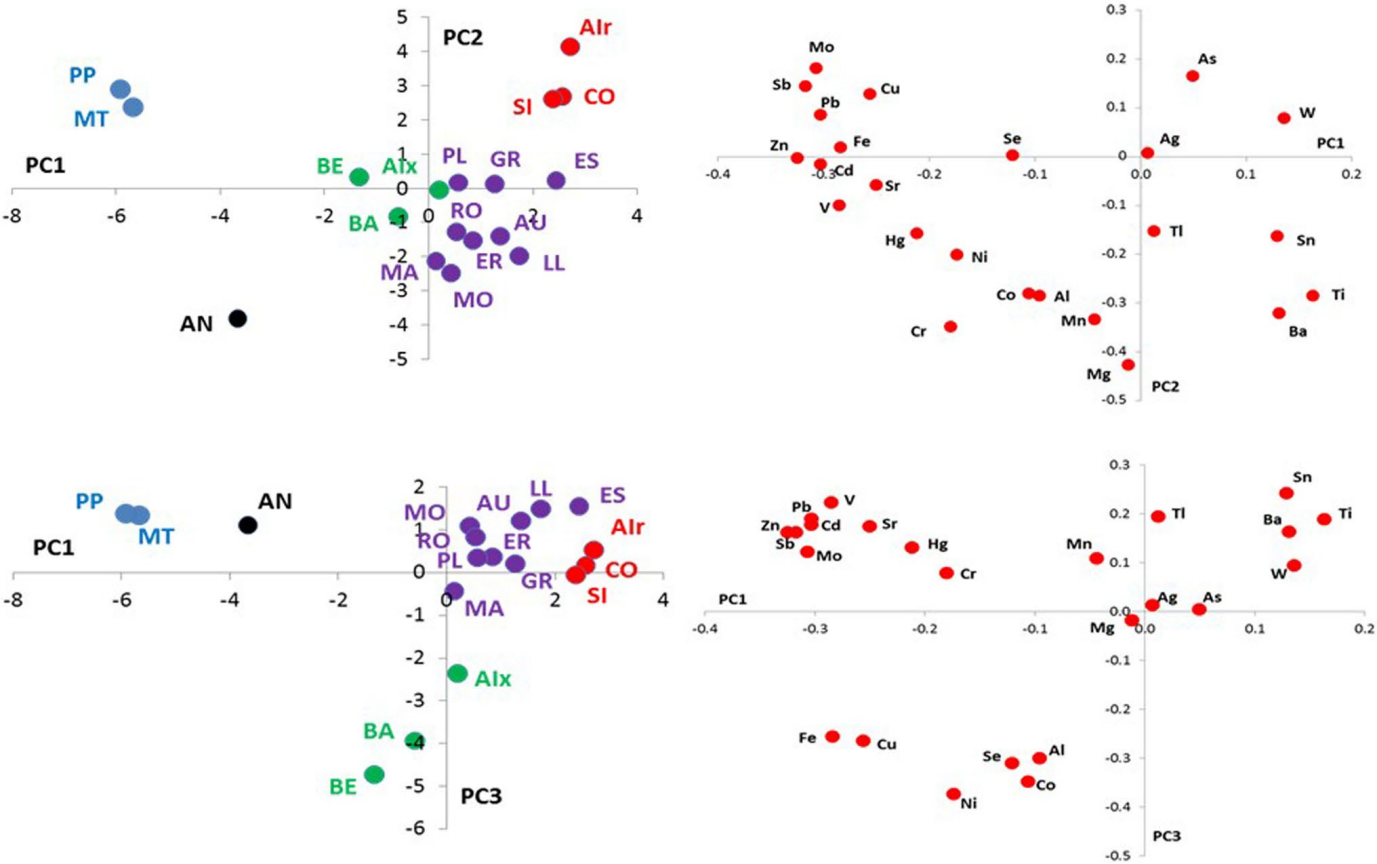
Scores and loadings plots (PC1–PC2 and PC1–PC3) obtained after PCA of the studied dataset

The structure of the data matrix was investigated by principal component analysis (PCA). This multivariate analysis method is widely used in environmental studies to obtain representative graphs to characterise the combined effect of different variables and recognise possible patterns, relationships, and correlations within data (Einax et al. 1998; Jolliffe 2002). Concentrations below the LOD were substituted by one-half of the LOD value for multivariate analysis. Data were centred and scaled before treatment when necessary. The appropriate number of principal components (PCs) used in the multivariate analysis was determined by a scree plot (data not shown). The selected model explains the 63% of the total variance by using three PCs (PC1 = 31%; PC2 = 19%; PC3 = 13%). PC1 presents positive loading values for As, W, Sn, Ti and Ba, all with potential lithogenic origin. PC2 my reflect the input from mining activities, and PC3 shows positive loading values for all the elements considered with the exception of Fe, Cu, Ni, Se, Co and Al (see Fig. 4).

Data processing was carried out by using the software The Unscrambler v. 9.7 (CAMO Software AS, Oslo, Norway) and R 3.2.2 (The R Foundation for Statistical Computing, USA) with Rstudio v. 1.0.143 (Rstudio Inc., USA).

## Results and discussion

### Metal and metalloid concentrations in surface sediments

The concentrations of metals and metalloids found in surface sediments of Pyrenean lakes are shown in Table 2, and in Fig. 2 in the form of Box-Whisker plots (after removing extreme and outlier values). Extreme concentrations were defined as *c* > Q_3_+3·(Q_3_–Q_1_), whereas outliers were identified as *c* > Q_3_+1.5·(Q_3_–Q_1_), being Q_1_ and Q_3_ the %25th and %75th percentiles, respectively (Otto 2007). The lake that shows extreme and/or outlier concentrations is somehow out from the “normality” defined by the rest of the lakes.

The elements have been classified in terms of concentration and abundance. In general, they follow this order: Al, Fe > Mg > Zn > Ti > Mn > As > Pb. Correlations significantly different from zero (*α* = 0.05, *r*_crit_ = 0.47) were found between Al-Fe (*r* = 0.78), Al-Zn (*r* = 0.50), Al-Mg (*r* = 0.58), Mg-Mn (*r* = 0.83), Mg-Ti (*r* = 0.53), Mg-Pb (*r* = 0.55) and Pb-Zn (*r* = 0.56), which corroborates the common natural origin (mostly lithologic) of the group constituted by Al, Fe, Ti and Mn (Bacardit and Camarero 2009). In this study, As was independent with respect to all other elements, and in fact, high concentrations of natural As in Pyrenean lake sediments are already well-known and reported by other authors (Camarero 2003; Catalan et al. 2006). Among the airborne trace elements with potential toxic effect, Pb is the one that may cause a greater impact in Pyrenean lake ecosystems (Bacardit and Camarero 2009). Indeed, the average concentration of Pb (146 mg·kg^−1^) found in the lake sediments investigated in this study is above the values reported (30–70 mg·kg^−1^) to produce toxicological effects (Del Valls and Chapman 1998).

Considering the individual concentrations of elements, some of them presented neither extreme nor outlier values (Al, Ba, Cr, Mg, Ni, Co, Hg, V and Ti) (Table 2). These results indicate relatively homogeneous concentrations of these elements within the surface sediments of the lakes considered in this study. Furthermore, the concentrations are of the same order than those reported in surface sediments from other Pyrenean lakes, such as Respomuso (2140 m) (Zaharescu et al. 2009), Légunabens (1655 m), Plan (2188 m) and Vidals d'Amunt (2684 m) (Bacardit et al. 2012). Consequently, concentration ranges (in mg·kg^−1^) of 24000 ± 9000 (Al), 66 ± 31 (Ba), 23.5 ± 7.8 (Cr), 4600 ± 1800 (Mg), 18 ± 11 (Ni), 480 ± 300 (Ti), 7.4 ± 4.1 (Co), 0.39 ± 0.19 (Hg) and 33 ± 12 (V) could be considered as representative for recent sediments of the Pyrenean lakes in igneous dominated watersheds. Additionally, these elements seems to be highly inter-correlated (Fig. 3), highlighting the correlations between Al-Ni (*r* = 0.70), Ba-Ti (*r* = 0.76), Cr-Mg (*r* = 0.76), Cr-V (*r* = 0.74 and Ni-Co (*r* = 0.87), consistent with general behaviour of these elements in Earth surface sedimentary environments (Hernandez et al. 2003).

Camarero (2003) measured the concentration of As, Cd, Cu, Hg, Pb, Se and Zn in surface sediments collected in 75 different Pyrenean lakes, a representative sample of an entire population of 1062 lakes larger than 0.5 Ha. Concerning As, Cu, Hg, Pb and Se, there is not a significant evidence that the samples used in Camarero’s study and those used in this study are drawn from different populations (Kruskal-Wallis test, *p* < 0.05). In the case of Cd and Zn, however, significantly higher concentrations were found in this study than in Camarero’s survey (samples collected at least 10 years before ours). The median concentration for Cd and Zn ranged from ~0.5 to ~1.0 mg·kg^−1^, and from ~120 to ~150 mg·kg^−1^, respectively. These two metals are highly correlated, especially when all data is considered (*r* = 0.99), but also after removing extreme values and outliers (*r* = 0.56), suggesting a common input source to lakes (Bing et al. 2016).

PCA of the dataset made up for all the surface sediments analysed was carried out in order to identify Pyrenean lakes with similar characteristics in terms of metal concentrations in recent sediments (Jolliffe 2002)*.* The scores and loadings plots over the first three PCs are shown in Fig. 4. According to this analysis, the studied Pyrenean lakes can be divided into 5 different groups depending on metal concentration in their surface sediments: (i) Siscar (SI), Comte (CO) and Airoto (Air); (ii) Montoliu (MT) and Pica Palomera (PP); (iii) Anglas (AN); (iv) Baiau (BA), Aixeus (Aix) and Bersau (BE), and (v) Aubé (AU), Estelat (ES), Gran del Pesso (GR), Eriste (ER), Llosas (LL), Mariola (MA), Monges (MO), Plan (PL) and Romedo de Dalt (RO). In general, the lowest element concentrations were found in sediments from lakes of the first group (SI, CO and air). As the PCA shows the exceptions are the extreme concentrations of As (1470 mg·kg^−1^) and W (4.96 mg·kg^−1^) found in the lake Airoto (Table 2). Air is located in the middle Pyrenees in a watershed dominated by igneous rocks. The natural richness in arsenopyrites (Bacardit and Camarero 2010b; Camarero 2003; Camarero et al. 2009; Zaharescu et al. 2009) and ferberite minerals of the area suggests that As and W are of lithogenic origin. Siscar (SI) and Comte (CO) are both metamorphic lakes and are located very close to each other, in the eastern part.

The second (Montoliu, MT and Pica Palomera, PP) and the third groups (Anglas, AN) are all located in the negative part of the PC1. Extreme concentrations of Fe, Cd, Mo, Pb, Sb, Cu and Zn were found in MT and PP caused surely by mining in these catchments (Camarero 2003). Anglas (AN, together with Bersau, BE) is the lake sited further west. Its sediments were significantly rich in Cd, Cr, Hg, Sr and Zn (Fig. 4). The exploitation of rich ores of mainly (but not only) Pb and Zn has been carried out since ancient times within the catchments of Montoliu (MT), Pica Palomera (PP) and Anglas (AN) lakes (Birch et al. 1996; Subias et al. 1999)*.* Thus, the extreme values found for those metals in the surface sediments of these lakes are probably related to a relatively recent mining activity within the lake catchments (Corella et al. 2017). The fourth group, formed by Baiau (BA), Aixeus (Aix) and Bersau (BE), includes lakes all located in sedimentary rocks. The three lakes presented significantly higher concentrations of Al, Co, Cu, Fe, Ni and Se in comparison with the rest of the lakes (Fig. 4). Regarding their geographical location Baiau (BA) and Aixeus and (Aix) are sited very closed to each other in the eastern part and Bersau (BE) is in the western. Therefore, this study was not able to corroborate the higher metal presence found in sediments from lakes in the eastern Pyrenees with respect to those in the western part in the range already reported (Camarero 2003), a fact that underlines the importance of the local conditions and characteristics of each specific area. The fifth group, including the half of the lakes studied, could be explained by the igneous lithology of the main part of the lakes included. All showed high concentrations of Ti, Mg, Ba, Mn, Sn and Tl (Table 2). The monzogranites and granodiorites are the major rock types exposed in this area (with the exception of Gran del Pesso, GR and Mariola, MA, which are sedimentary lakes, and Montoliu, MT, which is metamorphic). Finally, the origin of the extreme concentrations of Tl, Ag and Se found in surface sediments from Monges (MO), Plan (PL) and Bersau (BE) lakes respectively, remains unclear and is an issue, which deserves further investigation. It could be an artefact due to a sample contamination during collection, manipulation, storage or analysis of samples, or respond to any specific characteristic of the area, including the possibility of any punctual or diffuse source of pollution.

### Sediment cores: historical record of metal concentrations

Sediment cores were divided into 3.5-cm layers and a consequence, the resulting depth profiles could be so smoothed that historical information remain hidden in them (Camarero et al. 1998), which is due to relatively low vertical resolution of our core sampling. There are examples in the literature, however, where 2.4-cm layers have been used to investigate historical records of pollution (Bacardit et al. 2012). It should be considered, moreover, that the sedimentation rate could vary depending on the location of the lake, the topography of its watershed and internal lake depositional processes. All these factors may induce an important uncertainty in the estimation of the age of each core slide. In fact, another study has estimated that the accumulation rate in the first 30 mm of sediments in the Pyrenean lake Redó is of 0.23 mm per year (Camarero et al. 1998). Therefore, the upper 5-cm layer in the lake has been accumulated in the last 200–300 years. Estimations done with sediment cores from other Pyrenean lakes, such as Estanya or Basa de Mora, obtained an interval between 0.10 and 0.20 mm of sediment accumulation per year with some fluctuations depending on the slide analysed (Morellón et al. 2011).

In the following discussion, we only consider the sediment cores with at least 17 cm long, e.g., those collected in 11 Pyrenean lakes (Eriste, ER; Pica Palomera, PP; Plan, PL; Mariola, MA; Aixeus, AIx; Aubé, AU; Romedo de Dalt, RO; Baiau, BA; Siscar, SI; Comte, CO; and Estelat, ES). In addition, 12 elements were only considered (Cu, Cr, Pb, Zn, As, Cd, Ni, Hg, Co, Mn, Sn and Sb) since, for the rest of the elements, the concentrations found below the LOD were abundant (data not shown). Ti concentration was used to normalise data, since its origin has been reported to be natural in the Pyrenees (Camarero 2003; Catalan et al. 2006). The vertical profile with depth of the normalised concentration of each element can be seen in Fig. S1.

Some elements behaved similarly over all the sediment core profile. After the first 5 cm from the surface, concentrations of Cd, Pb, Sb and Sn considerably decreased. This trend is observed in 8 lakes, e.g., Eriste (ER), Plan (PL), Mariola (MA), Aixeus (Aix), Aubé (AU), Romedo de Dalt (RO), Baiau (BA) and Estelat (ES), with the following exceptions: Pb in Aixeus (Aix) and Pb and Sn in Baiau (BA). In all these lakes, the concentrations of elements such as Cd, Pb, Sb and Sn have risen notably in recent years. According to the estimation done by Camarero et al. (1998), this observed rise would have started approximately with the industrial revolution (Bacardit et al. 2012; Camarero et al. 1998).

Apart from the general trend that was observed for Cd, Pb, Sb and Sn, the highest concentrations for the rest of the elements were observed at a depth of around 15 cm (~1300 to 1400 years old, according to the sedimentation rate estimated by Camarero et al. 1998). This fact is not something unusual. Wars, large-scale fires, climate changes, mining activity, economic transition and bans on certain chemicals have been observed in sediment cores from Pyrenean lakes (Bacardit et al. 2012; Camarero et al. 1998; Camarero et al. 2009; Corella et al. 2014; Corella et al. 2017; Corella et al. 2018; Farmer et al. 2015; González-Sampériz et al. 2017; Larrasoaña et al. 2010; Morellón et al. 2009; Moreno et al. 2012; Morra et al. 2015; Trapote et al., 2018; Vegas-Vilarrúbia et al., 2018). For instance, the analysis of potential harmful trace elements (PHTE; as Pb, Hg, Zn, As and Cu) in sediments form Lake Marboré (Central Pyrenees) showed a common pattern related to mining and metalworking activities recorded in other European areas, with intensification peaks during Roman period, Medieval times and Industrial revolution (Corella et al. 2021). Moreover, the geochemical analyses carried out in a sediment core collected in Lake Montcortés (Pre-Pyrenees) allowed the reconstruction of Hg and Pb atmospheric deposition over the past seven centuries in the Pyrenees (Corella et al. 2017). In the case of Hg, it was possible to conclude that volcanic eruptions may have been responsible for some Hg flux peaks recorded in sediments from medieval times. During the Preindustrial period (CE, 1550–1840), the Hg production from the two largest mercury mines in the world, located in southern Spain (Almaden mine) and Slovenia (Idrija mine), together with climatological conditions, may intensify the Hg deposition in the lake.

Metal and metalloid records are not showing a decline in the most recent sediments which would be expected because of the massive reduction in emissions since the 1970s (Rose et al. 2012). This effect could be hidden since our surface slice (1.5 cm) accounts probably for the last 65 years.

The case of Plan (PL, located in the middle Pyrenees) lake is worth mentioning, as it shows the same tendency in all the metals and metalloids considered, except for Hg and Zn. The first 5-cm layer exhibits the highest concentrations of most elements and the concentration decreases downwards. However, the local maxima of Hg and Zn are found at 5–10-cm depth and at 25–30 cm. Open-air mining activities in this area reached a maximum in the Middle Ages, and this could explain the presence of these peaks of concentration in deeper sediments (Corella et al. 2017).

However, high concentrations of metals and metalloids by themselves may not necessarily reflect contamination, since natural metal concentrations in lake sediments can fluctuate markedly (Koinig et al., 2003) and atmospheric metal deposition must therefore be extremely high to create an observable disturbance in the system. Sedimentation characteristics of each lake are crucial in this sense. Sediment remobilisation by water currents and/ or gravitational waves causes heterogeneities in the sedimentary sequences of the lakes. The morphological characteristics of the lake and the watershed may also have an important role in the trace metal deposition on lacustrine sediments. Finally, differences in altitude across the Pyrenees cause climate gradients that may delimit trace metal deposition on lake surfaces (Corella et al. 2018). Thus, contamination may be determined by both, the concentration and the particular sedimentation characteristics of each lake. Accordingly, the use of the enrichment factors (EF) is a more appropriate way to assess the contamination impact in each specific basin.

### Enrichment factors: anthropogenic inputs

The enrichment factor (EF) is a good indicator of pollution since it reflects consistent distribution pattern of a pollutant. Moreover, this index allows us to discern between natural and anthropogenic sources, via comparison of surface sediments and the deepest parts of the cores (Camarero 2003). For this purpose, the EFs corresponding to the upper 5 layers (0–1.5, 1.5–5.0, 5.0–8.5, 8.5–12.0, 12.0–15.5 cm) of each core were calculated.

To calculate the EF, element concentrations were normalised with those of Ti (Eq. 1), since its origins has been reported to be natural in the Pyrenees (Camarero 2003; Catalan et al. 2006) and because Ti is virtually immobile in lake sediments (Böes et al. 2011).

1 $$E{F}_M=\frac{C_M/{C}_{Ti}}{C_M^0/{C}_{Ti}^0}$$

*M* stands for the studied element, *C* represents the element concentration in the sediment sample, and *C*^0^ is the estimated background value for that element on each area (Camarero et al. 1998). In this case, the reference metal level *C*^0^ was defined as the concentration measured in the bottom sample of each core.

An EF value higher than 2 shows non-natural input of the element and indicates the presence of anthropogenic pollution (Camarero 2003). Despite the concentration of some elements was rather high (Al, Mg, Ti and V), some of the elements (Ag, Al, Cr, Hg, Mg, Mo, Se, Sr, Tl, V and W) exhibited EF values lower than 2 in all the lakes investigated. As such, the low EFs obtained for Ag, Al, Cr, Hg, Mg, Mo, Se, Sr, Tl, V and W imply essentially natural origin of these elements in high-altitude lacustrine ecosystems of the Pyrenees.

The elements that present a value of EF above 2 in at least one lake are included in Table 3, where the average, maximum and minimum values of EF per element and lake are shown. Only three lakes (Aixeus, AIx, Eriste, ER and Comte, CO) present mean EFs below 2 for all the elements studied. In other words, more than the 80% of the lakes showed values of EF above 2 for at least one of the elements investigated in at least one core layer, which corroborates the existence of historical anthropogenic inputs of elements in the studied area, as already reported in previous works (Catalan et al. 2006). The case of Pica Palomera (PP) is the most remarkable, as its average EFs for Cd, Cu and Zn were far above 2. The lakes Aubé (AU) and Mariola (MA), on the one hand, and Estelat (ES), Plan (PL), Pica Palomera (PP), Romedo de Dalt (RO) and Siscar (SI), on the other, also present rather high mean values of EF for Cd and Pb, respectively. The average EFs found in Estelat (ES), Plan (PL) and Romedo de Dalt (RO), for Sn and Sb were also quite high. The presence of all these elements in the past is evident all over the Pyrenees and, among them, Pb is considered to be as one of the most important pollutant across Europe, with a median overall EF of 2.3 (Camarero et al. 2009).

**Table 3:** EF values calculated for upper layers (0–1.5, 1.5–5.0, 5.0–8.5, 8.5–12.0, 12.0–15.5 cm) of the sediment cores in some Pyrenean lakes. The average, minimum and maximum values of the EFs calculated are provided

Lake	As	Ba	Cd	Co	Cu	Fe
AIx	0.70 (0.20–1.3)	1.0 (0.90–1.0)	–	0.90 (0.60–1.7)	0.700 (0.40–0.80)	0.70 (0.40–0.90)
AU	1.3 (0.80–2.4)	1.1 (1.0–1.2)	2.4 (<DM–6.2)	1.2 (0.80–1.8)	1.2 (0.90–1.3)	1.1 (0.80–1.4)
BA	0.60 (0.1.0–1.2)	1.2 (0.90–2.2)	1.4 (0.30–3.1)	0.60 (0.20–0.90)	0.70 (0.10.–1.3)	0.80 (0.20–1.7)
CO	0.90 (0.60–1.2)	1.5 (1.0–2.2)	1.2 (<DM–2.2)	0.90 (0.50–1.1)	1.1 (0.90–1.3)	0.90 (0.80–1.1)
ER	0.70 (0.50–0.90)	1.1 (1.0–1.3)	–	1.0 (0.90–1.1)	1.0 (0.90–1.1)	0.90 (0.80–1.1)
ES	1.0 (0.40–1.4)	1.3 (0.90–1.8)	–	1.0 (0.80–1.2)	2.2 (0.50–7.2)	1.0 (0.80–1.2)
MA	0.90 (0.50–1.4)	1.2 (1.0–1.4)	2.4 (<DM–7.0)	0.90 (0.50–1.3)	0.80 (0.60–1.0)	0.80 (0.60–1.1)
PL	1.3 (0.40–3.3)	1.1 (0.70–1.6)	–	1.1 (<DM–2.5)	1.1 (0.60–2.0)	1.4 (0.40–2.8)
PP	0.70 (0.40–1.1)	1.0 (0.80–1.2)	22.6 (8.2–34)	1.4 (0.70–2.3)	3.4 (1.3–5.2)	0.60 (0.40–1.1)
RO	1.0 (0.60–1.3)	1.1 (0.90–1.5)	–	1.1 (0.80–1.3)	1.0 (0.70–1.2)	1.1 (0.80–1.5)
SI	1.1 (0.70–1.3)	1.3 (0.90–2.0)	–	1.1 (1.0–1.3)	1.1 (0.90–1.2)	1.6 (0.30–1.7)
Lake	Mn	Ni	Pb	Sb	Sn	Zn
AIx	1.0 (0.90–1.2)	0.80 (0.60–0.90)	1.0 (0.80–1.3)	1.2 (0.90–1.6)	0.90 (0.80–1.1)	0.90 (0.60–1.2)
AU	1.0 (0.70–1.3)	1.0 (0.80–1.3)	1.6 (1.3–1.8)	2.3 (1.1–4.3)	1.6 (1.1–2.8)	1.2 (1.1–1.3)
BA	1.0 (0.60–1.9)	0.70 (0.20–1.0)	1.3 (0.60–2.5)	1.1 (0.50–1.8)	2.2 (1.0–5.8)	0.80 (0.60–1.0)
CO	1.0 (0.80–1.1)	0.90 (0.70–1.1)	1.7 (1.1–2.0)	–	1.4 (1.0–1.7)	1.0 (0.80–1.3)
ER	0.90 (0.80–1.0)	0.90 (0.80–1.0)	1.9 (1.0–2.8)	1.4 (<DM–3.0)	1.2 (0.40–1.8)	0.90 (0.90–1.1)
ES	1.0 (0.80–1.1)	0.80 (0.50–1.0)	2.4 (0.70–5.8)	4.3 (<DM–19)	1.7 (1.0–3.3)	1.2 (0.70–1.8)
MA	0.80 (0.50–1.1)	0.70 (0.40–1.1)	1.7 (1.1–2.9)	2.1 (1.0–4.8)	2.8 (1.0–7.6)	0.80 (0.40–1.2)
PL	1.3 (0.70–2.4)	1.7 (0.10.–3.9)	5.0 (0.60–11.)	13.8 (<DM–44)	2.0 (0.80–3.7)	0.60 (0.40–1.1)
PP	0.70 (0.50–1.1)	0.70 (0.40–1.0)	2.2 (1.4–2.8)	0.90 (0.60–1.8)	1.3 (0.70–1.8)	14 (4.7–27)
RO	1.0 (0.80–1.2)	1.0 (0.80–1.3)	2.0 (0.90–4.6)	5.2 (0.80–20)	2.1 (0.90–6.5)	1.0 (0.80–1.3)
SI	0.90 (0.70–1.0)	1.2 (1.0–1.6)	2.4 (0.90–4.0)	–	1.3 (0.80–2.1)	1.2 (1.0–1.6)

**Fig. 5:**
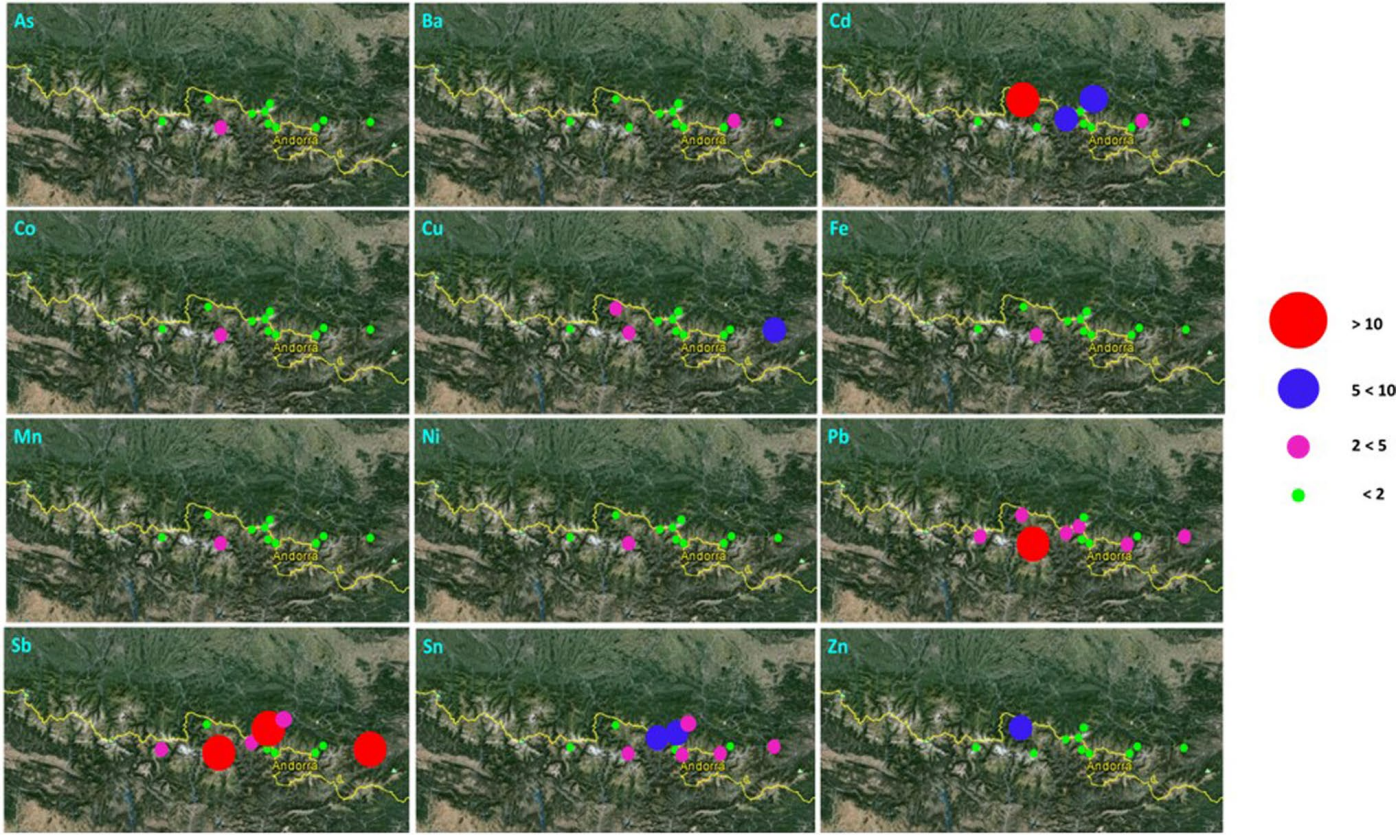
EF values calculated for surface sediments in different Pyrenean lakes

To assess the anthropogenic inputs over recent years, the EFs calculated for the surface sediments (0–1.5 cm) can be used (Fig. 5). Given the low sedimentation rate observed in alpine lakes (Appleby 2000; Böes et al. 2011; Camarero et al. 1998), we might expect to account in this layer for the contamination of the last 20–65 years.

Cd, Cu, Pb, Sb and Sn are the elements that showed the highest EFs in surface sediments, and the ones that have the widest geographical distribution (Fig. 5). It is to be highlighted that the lake Aixeus, (Aix) did not show EFs above 2 in any of the studied elements, showing that the recent anthropogenic input of metals and metalloids in this area is negligible. However, in Plan (PL), EFs above 2 has been obtained for elements such as As, Co, Cu, Fe, Mn, Ni, Pb, Sb and Sn. Furthermore, in the case of Sb, this lake has shown the highest value of EF (31.4). The antimony mines located in the Ribes Valley (Catalonia, Eastern Pyrenees) could have influenced on this area. These mines were exploited at the end of the nineteenth century and the beginning of the twentieth. Actually, accumulation of antimony and other potentially toxic elements in plants around the mine was reported. The mines closed around 1960 (Bech et al. 2012).

These results seem to indicate the presence of a local pollution source (probably related to mining activities and the use of fossil fuels) that, together with the long-range transport, may cause the accumulation of these elements in high-altitude lake sediments, mainly dependent on the dominant Atlantic winds (from west to east), and the specific orography of each basin (Hernandez et al. 2003).

## Conclusions

Technical and scientific advances have allowed the humankind to gain the power to exert environmental modifications at a planetary scale. However, the changes do not always positively affect the ecosystems, as they alter significantly the environment through different forms of pollution. This study confirms that the influence of human activity in pre-industrial and modern sediments from the lakes studied is, although significant, relatively low.

Taking into account the limitations that having sliced the sediment cores into 3.5-cm layers, the main conclusions of this study are in good agreement with those obtained in other similar works (Camarero et al. 1998; Camarero et al. 2009). Metal and metalloid concentrations are, in general, rather low, with values of EF below 2 except for Cd, Pb, Sb and Sn. The traditional mining activities which started long time ago (probably in Roman times) and the massive use of fossil fuels and industrial activity over the last decades have impacted, to a greater or lesser extent, the Pyrenean lake ecosystems. Metals released into the atmosphere via industrial activities can reach remote mountain areas and, consequently, high-altitude lakes, by long-range atmospheric transport. At the same time, natural contributions should not be neglected. Thus, the lithogenic composition of the lake basin must be taken into consideration to make a correct interpretation of the accumulation of some elements (such as As and Ti) in lake sediments.

Overall, the comparison of the sediment composition between lakes of the same district is not straightforward since each lake basin has distinct behaviours and pollution footprints related to its geographical position (relative to pollution sources), orographic characteristics, climatic features or in-lake depositional processes.

There is a need for further long-term monitoring studies that should further incorporate not only benthic studies, but also water-column profiles, and they should be extended to a representative number of lakes in order to create an ambitious monitoring network, which would enable the observation of long-time series and the study of both regional and global trends.

## Supplementary information

Supplementary file 1 **Fig. S1**. Vertical normalised concentration profiles of the elements measured in the sediment cores obtained from Pyrenean lakes. Depth in cm is plotted in axes Y. Concentrations normalised with Ti are plotted in axes X

## Data Availability

Upon request authors should be prepared to send relevant documentation or data in order to verify the validity of the results presented.
