# Supplementary material for: Metals and metalloids in high-altitude Pyrenean lakes: sources and distribution in pre-industrial and modern sediments
Source: Environ Sci Pollut Res Int. 2023 Jul 10;30(37):87561–74. doi: 10.1007/s11356-023-28347-6 (PMC10406682; doi:10.1007/s11356-023-28347-6)
Supplement: Supplementary file 1 — Fig. S1. Vertical normalised concentration profiles of the elements measured in the sediment cores obtained from Pyrenean lakes. Depth in cm is plotted in axes Y. Concentrations normalised with Ti are plotted in axes X [file 11356_2023_28347_MOESM1_ESM.pdf]

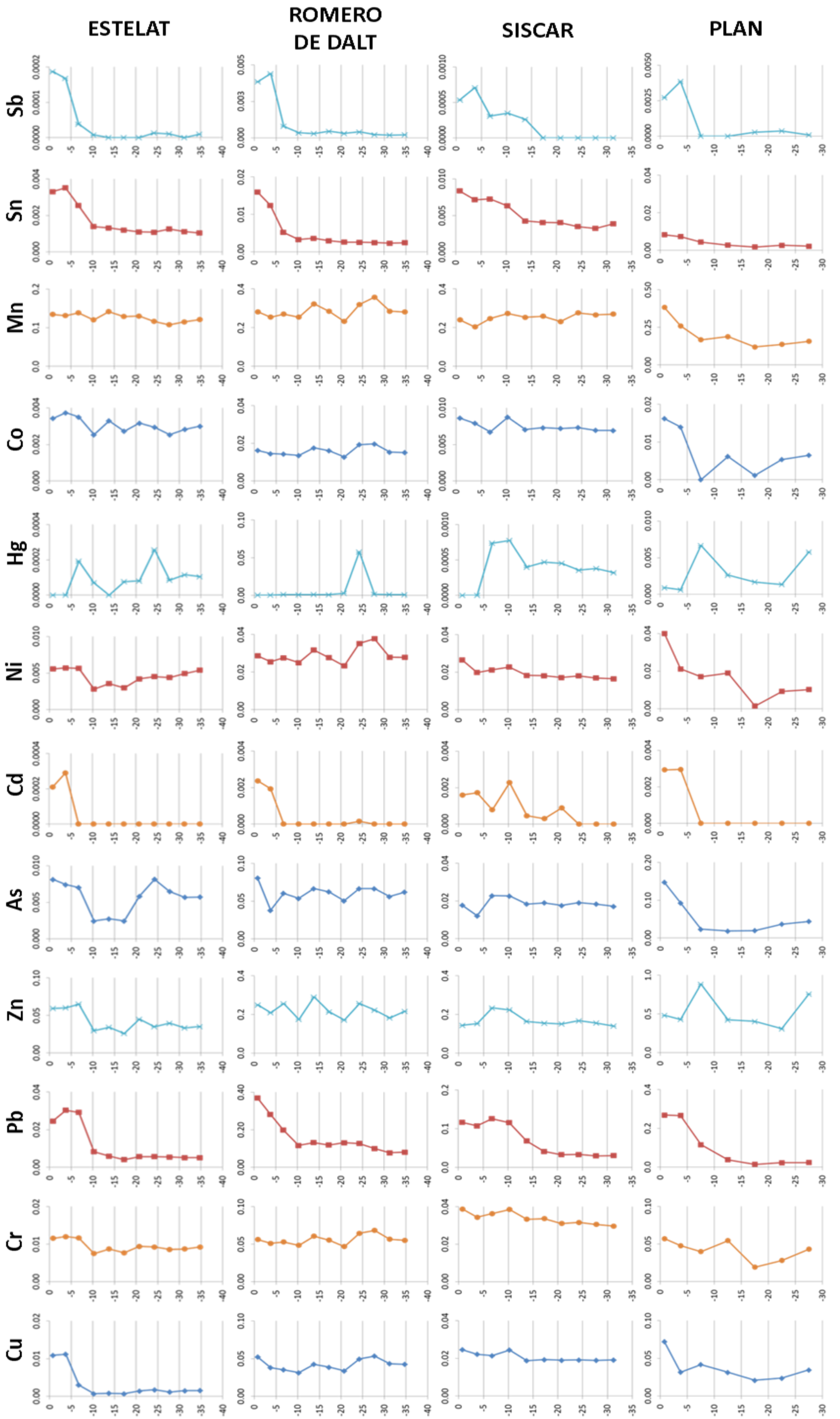

Figure S1. Vertical concentration profiles of the elements measured in the sediment cores obtained from Pyrenean lakes. Depth in cm is plotted in axes Y. Concentrations normalised with Ti are plotted in axes X.

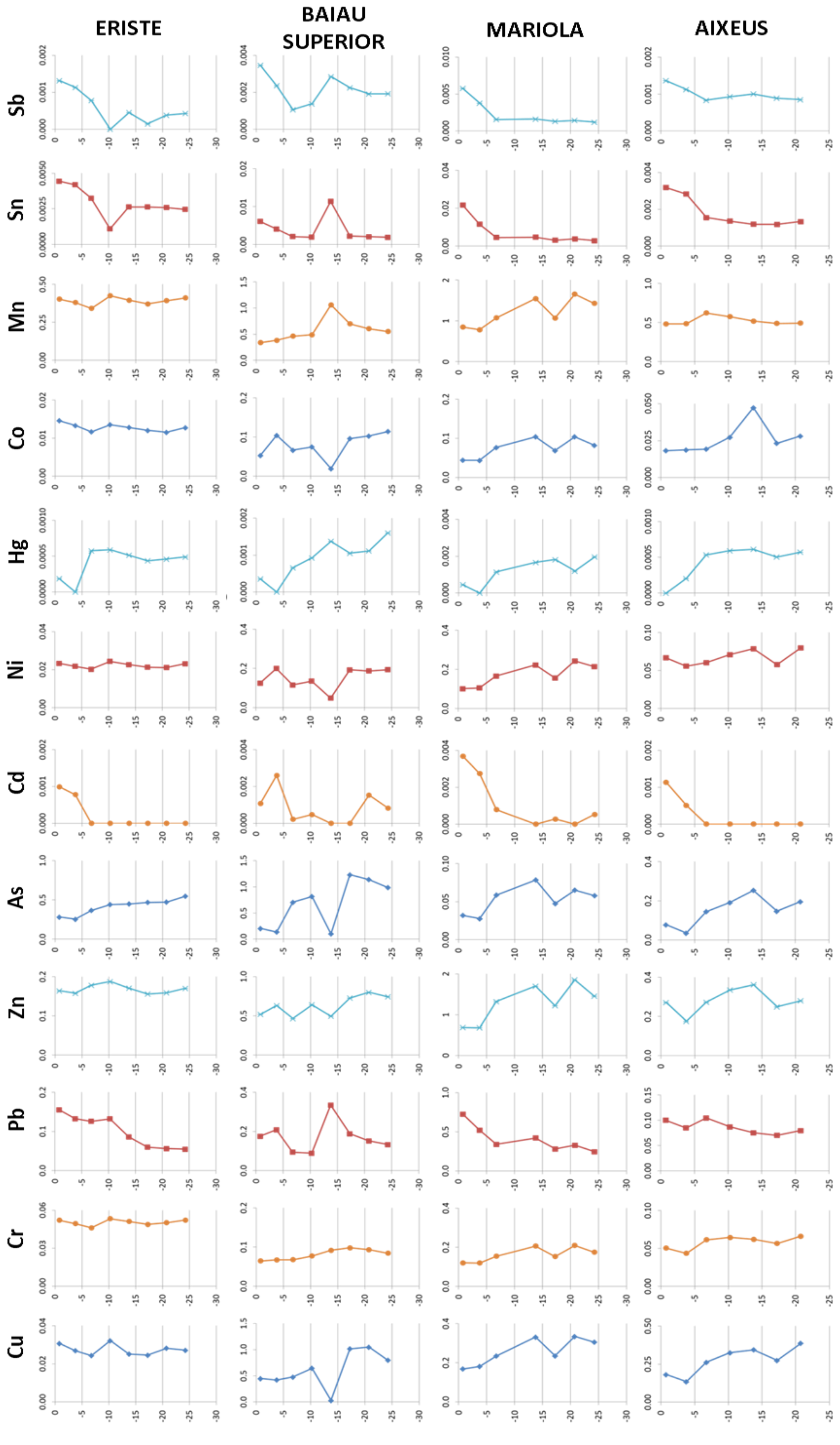

Figure S1 (cont.). Vertical concentration profiles of the elements measured in the sediment cores obtained from Pyrenean lakes. Depth in cm is plotted in axes Y. Concentrations normalised with Ti are plotted in axes X.

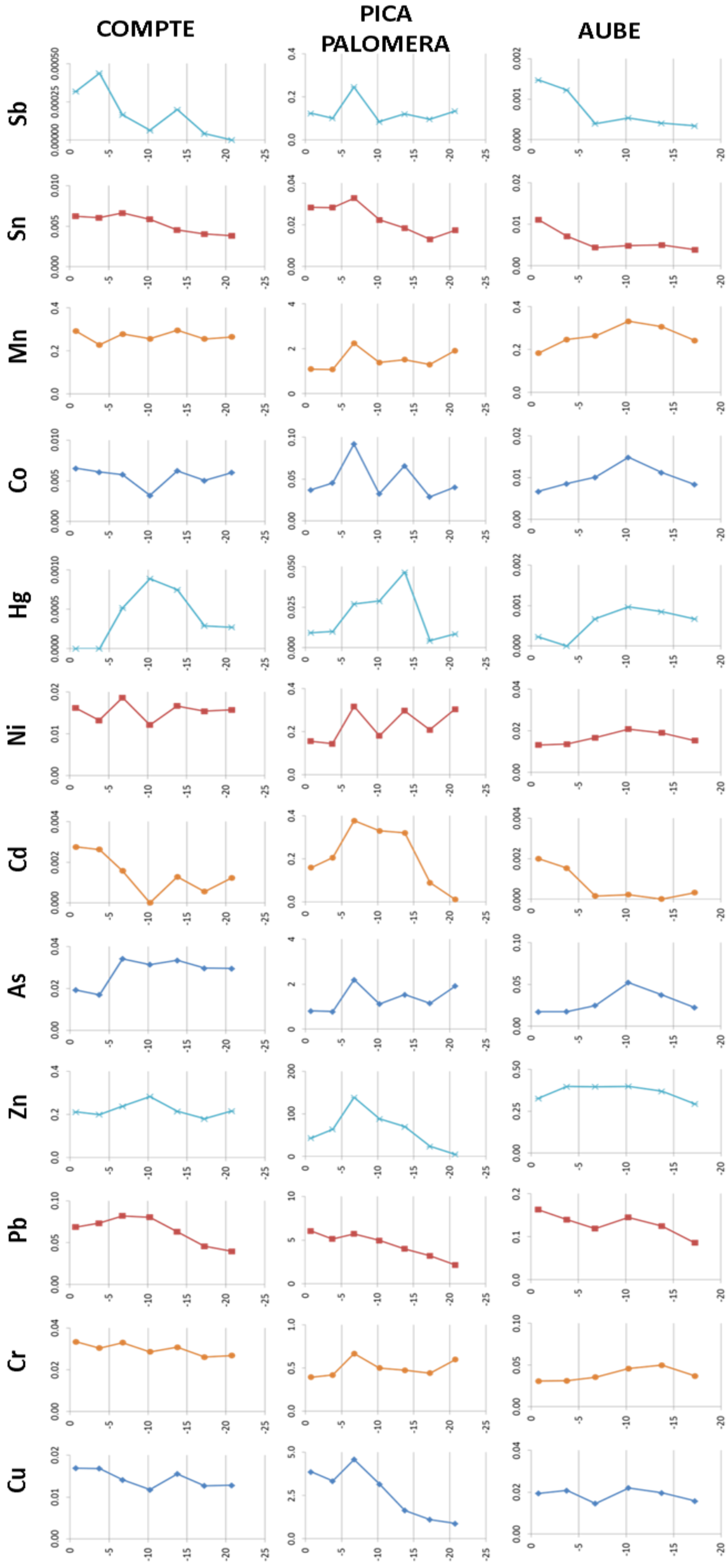

Figure S1 (cont.). Vertical concentration profiles of the elements measured in the sediment cores obtained from Pyrenean lakes. Depth in cm is plotted in axes Y. Concentrations normalised with Ti are plotted in axes X.
